# Supplementary material for: Comparative Virucidal Activities of Essential Oils and Alcohol-Based Solutions against Enveloped Virus Surrogates: In Vitro and In Silico Analyses
Source: Molecules. 2023 May 18;28(10):4156. doi: 10.3390/molecules28104156 (PMC10220882; doi:10.3390/molecules28104156)
Supplement: Supplementary file 1 [file molecules-28-04156-s001.zip › molecules-2372938-supplementary.pdf]

Supplementary Materials

Comparative Virucidal Activities of Essential Oils and Alcohol-Based Solutions against Enveloped Virus Surrogates: In Vitro and In Silico Analyses

Valentina Parra-Acevedo [1], Raquel E. Ocazionez [1,*], Elena E. Stashenko [1], Lina Silva-Trujillo [1] and Paola Rondón-Villarreal [2]

[1] Centro de Cromatografía y Espectrometría de Masas—CROM-MASS, Universidad Industrial de Santander, Bucaramanga 680002, Colombia; valentina2218470@correo.uis.edu.co (V.P.-A.); elena@tucan.uis.edu.co (E.E.S.); lina2198192@correo.uis.edu.co (L.S.-T.)

[2] Facultad de Ciencias Médicas y de la Salud, Instituto de Investigación Masira, Universidad de Santander, Bucaramanga 680003, Colombia; diseno.molecular@udes.edu.co

* Correspondence: relocaz@uis.edu.co

Table S1. AutoDock Vina binding energies values of compounds identified in the EO blend for target proteins.

Type	Compound	DENV-2		CHKV
		E	prM/M	E1-E2-E3
Sesquiterpene hydrocarbons	δ-Cadinene	−8.61 ± 0.8	−5.47 ± 0.1	−6.25 ± 0.1
	α-Guaiene	−8.55 ± 1.0	−5.29 ± 0.1	−6.38 ± 0.2
	γ-Cadinene	−8.22 ± 1.0	−5.26 ± 0.2	−6.22 ± 0.1
	α-Bulnesene	−8.12 ± 0.9	−5.37 ± 0.2	−6.26 ± 0.2
	Germacrene D	−7.03 ± 1.1	−5.40 ± 0.1	−6.25 ± 0.2
	β-Elemene	−6.49 ± 0.9	−4.85 ± 0.5	−5.92 ± 0.2
	trans-β-Caryophyllene	−6.48 ± 0.5	−5.36 ± 0.2	−6.32 ± 0.1
	α-Muurolene	−5.91 ± 0.5	−5.26 ± 0.3	−6.18 ± 0.2
	γ-Muurolene	−5.91 ± 0.3	−5.26 ± 0.3	−6.18 ± 0.2
	α-Humulene	−5.90 ± 0.5	−5.29 ± 0.3	−6.37 ± 0.3
Oxygenated sesquiterpenes	*epi*-α-Muurolol	−8.10 ± 1.0	−5.42 ± 0.1	−6.37 ± 0.2
	Farnesol *	−6.57 ± 0.6	−5.33 ± 0.2	−6.01 ± 0.2
	α-Cadinol *	−6.54 ± 0.5	−5.28 ± 0.2	−6.70 ± 0.2
	α-Eudesmol *	−6.50 ± 0.6	−5.47 ± 0.1	−6.70 ± 0.3
	Elemol	−6.49 ± 0.9	−5.02 ± 0.2	−5.90 ± 0.2
	Germacrene D-4-ol	−5.80 ± 0.6	−5.41 ± 0.1	−6.38 ± 0.2
	epi-α-Cadinol	−5.75 ± 0.6	−5.18 ± 0.1	−6.29 ± 0.2
	trans-Nerolidol	−5.70 ± 0.5	−4.62 ± 0.2	−5.34 ± 0.3
	Patchoulol	−5.67 ± 0.2	−4.97 ± 0.1	−6.45 ± 0.2
	Caryophyllene oxide	−5.90 ± 0.5	−5.25 ± 0.1	−6.50 ± 0.3
Monoterpene hydrocarbons	Limonene	−7.22 ± 0.2	−4.60 ± 0.4	−5.16 ± 0.1
	γ-Terpinene	−7.18 ± 0.4	−4.95 ± 0.4	−5.29 ± 0.1
	p-Cymene	−7.16 ± 0.5	−4.89 ± 0.3	−5.34 ± 0.1
	trans-β-Ocimene	−6.47 ± 0.3	−4.62 ± 0.1	−4.81 ± 0.1
	β-Myrcene	−5.98 ± 0.6	−4.08 ± 0.2	−4.72 ± 0.2
	α-Pinene	−5.02 ± 0.3	−4.24 ± 0.1	−5.13 ± 0.1
Phenolic compounds	Carvacrol	−7.32 ± 0.3	−5.29 ± 0.4	−5.49 ± 0.1
	Thymol	−7.03 ± 0.4	−5.31 ± 0.3	−5.48 ± 0.1
	Eugenol *	−6.54 ± 0.5	−5.16 ± 0.4	−5.46 ± 0.3
Oxygenated monoterpenes	Isopulegol *	−6.70 ± 0.6	−4.85 ± 0.2	−5.19 ± 0.1

	Citronellyl acetate	−6.32 ± 0.4	−4.81 ± 0.2	−5.40 ± 0.2
	Geranyl acetate	−6.30 ± 0.5	−5.23 ± 0.2	−5.71 ± 0.2
	Neral	−6.25 ± 0.5	−4.10 ± 0.2	−4.86 ± 0.2
	Geranial	−6.20 ± 0.4	−4.28 ± 0.2	−4.95 ± 0.2
	Citronellol	−6.07 ± 0.3	−4.51 ± 0.2	−5.01 ± 0.1
	Neryl hexanoate	−5.98 ± 0.4	−5.24 ± 0.2	−5.71 ± 0.3
	Geraniol	−5.91 ± 0.5	−4.65 ± 0.2	−5.18 ± 0.1
	Citronellal	−5.75 ± 0.5	−4.03 ± 0.3	−4.72 ± 0.2
	Linalool	−5.72 ± 0.5	−4.31 ± 0.2	−4.91 ± 0.1
	1,8-Cineole	−4.77 ± 0.2	−4.24 ± 0.2	−5.16 ± 0.2
Other oxygenated compounds	*n*-Decanal	−5.47 ± 0.3	−5.47 ± 0.3	−4.56 ± 0.1
	6-Methyl-5-hepten-2-one	−5.51 ± 0.2	−4.31 ± 0.2	−4.43 ± 0.2

* Compounds marked with an asterisk were identified in the present study, and the other compounds were also identified in a previous study [25].

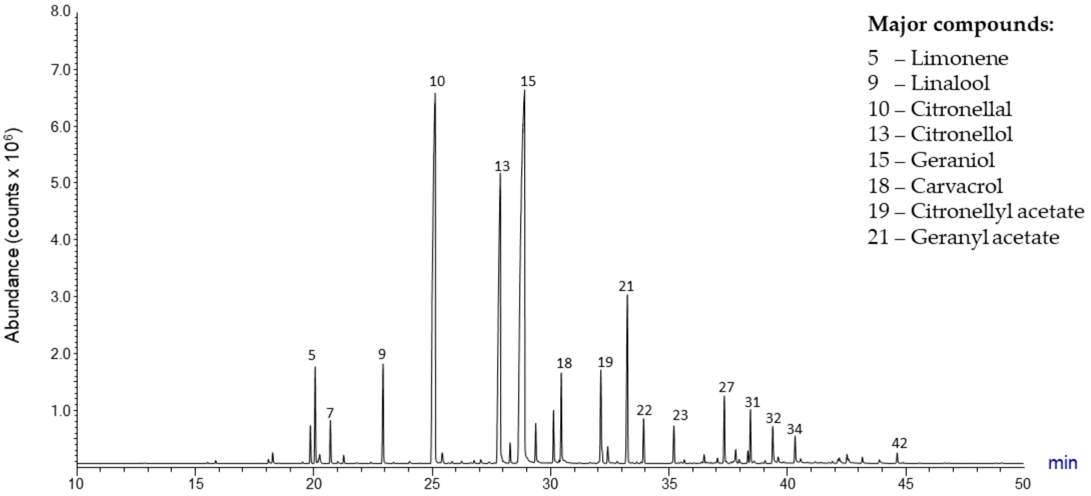

Figure S1. Chromatogram of the blend of seven essential oils used for virucidal activity study. DB-5 capillary column (60 m). Split 1:30. See peak identification in Table 5.
